# Label‐Free and Immobilization‐Free Protein‐Binding Assays by Ultraviolet Transient Absorption Microscopy

**DOI:** 10.1002/advs.202519985

**Published:** 2026-01-27

**Authors:** Jianghao Shen, Qiangqiang Wang, Fan Wu, Yang Gao, Lu Lan, Pu Wang

**Affiliations:** ^1^ Institute of Medical Photonics, Beijing Advanced Innovation Center for Biomedical Engineering, School of Biological Science and Medical Engineering Beihang University Beijing China; ^2^ Vibronix Inc. West Lafayette Indiana USA

**Keywords:** label‐free detection, protein‐ligand interactions, ultraviolet transient absorption

## Abstract

Protein–ligand interactions are central to understanding biological mechanisms and drug discovery, yet conventional assays often rely on labeling or immobilization that can alter natural binding. Here, we introduce ultraviolet transient absorption microscopy (UV‐TAM), which directly detects binding through ligand‐induced changes in the excited‐state dynamics of tryptophan residues. Using a femtosecond deep‐UV pump and a near‐UV probe, UV‐TAM enables label‐free, in‐solution measurements with only microliter sample volumes. We demonstrate its capability using plasma proteins—bovine serum albumin and hemoglobin—with alkaloid ligands, berberine and palmatine. Binding events are clearly identified through time‐resolved spectral changes. Quantitative analysis of hemoglobin–alkaloid interactions yields dissociation constants in close agreement with isothermal titration calorimetry. UV‐TAM thus provides a robust, calibration‐free platform for studying protein interactions in solution, with significant potential for biochemical research and high‐throughput drug discovery.

## Graphical abstract

A femtosecond UV pump–probe microscope resolves binding‐induced changes in protein excited‐state dynamics and converts these intrinsic contrasts into an equilibrium affinity readout. By probing tryptophan responses directly in solution, the approach enables quantitative determination of dissociation constants without labeling or immobilization, offering a minimally perturbative platform for protein–ligand interaction analysis.

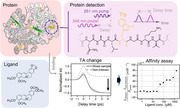

## 1 Introduction

Many critical aspects of cellular function, including signal transduction, enzymatic regulation, and molecular recognition, depend on the specific binding between proteins and their partners. Accurate quantification of these interactions under physiologically relevant conditions is essential for both basic biochemical research and drug discovery. Despite substantial progress in interaction analysis techniques, existing mainstream methods face notable limitations. Techniques such as surface plasmon resonance (SPR) [1] and biolayer interferometry (BLI) [2] rely on surface immobilization, which may perturb the native binding environment and introduce steric hindrance [3]. Fluorescence‐based methods, including differential scanning fluorimetry (DSF) [4] and microscale thermophoresis (MST) [5], often require chemical labeling, potentially altering protein conformation or binding affinity.

To avoid perturbing native binding events in solution, methods free of immobilization and labels are required. One approach is to exploit the intrinsic fluorescence as a readout (e.g., label‐free MST) [6], but its applicability is constrained by low fluorescence quantum yields. Other label‐free techniques, such as isothermal titration calorimetry (ITC) [7] and backscattering interferometry (BSI) [8], bypass fluorescence altogether by monitoring physical properties of the solution. However, these methods are limited by their dependence on specific binding‐induced changes—ITC requires measurable enthalpy shifts, while BSI depends on detectable refractive index changes. Another technique—kinetic capillary electrophoresis–mass spectrometry (KCE–MS) [9]—offers an alternative strategy by distinguishing bound and unbound species based on differences in electrophoretic mobility. However, kinetic capillary electrophoresis requires at least one interaction partner to carry a significant net charge, limiting its applicability. Consequently, kinetic capillary electrophoresis–mass spectrometry is mainly used to measure interactions involving charged biomolecules with relatively weak affinities. The method also suffers from limited resolution for small‐molecule ligands, complex instrumentation requirements, low reproducibility, and cumbersome workflows.

To overcome these limitations, we developed a label‐free and immobilization‐free detection method based on femtosecond ultraviolet transient absorption microscopy (UV‐TAM), in which a UV pump pulse selectively excites tryptophan residues in proteins (the primary electron donors), enabling sensitive and direct probing of their excited‐state dynamics in solution without relying on fluorescence or significant solution property changes. Using BSA and Hb as binding targets, and the alkaloids (berberine and palmatine) as ligands for validation, binding events were clearly distinguished through differences in excited‐state lifetimes. Using a combination of phasor analysis [10] and the law of mass action, the dissociation constants (*K*_D_) of the Hb–alkaloid interactions were quantified. The calculated *K*_D_ values were in strong agreement with those obtained from ITC [11], confirming the method's ability for quantitative interaction analysis.

## 2 Results

### 2.1 Principle of UV‐TAM for Protein‐Ligand Interaction Measurement

In a typical stimulated emission transient absorption (TA) experiment, a pump laser pulse excites molecules from the ground state to an excited state. After a controlled delay time (Δ*t*), a probe pulse stimulates the excited molecules down to the ground state and generates new coherent photons identical to those in the original probe pulse. By monitoring changes in the probe intensity, the population dynamics of excited states can be captured, offering a quantitative read‐out of the excited‐state kinetics [12]. After excitation, the proteins with undetectable fluorescence (e.g., Hb) return to the ground state mainly via non‐radiative decay rather than spontaneous emission (i.e., fluorescence). However, in TA microscopy, the stimulating field of the femtosecond probe pulse can transiently compete with non‐radiative decay, revealing the excited‐state dynamics even in non‐fluorescent proteins [13].

**FIGURE 1:**
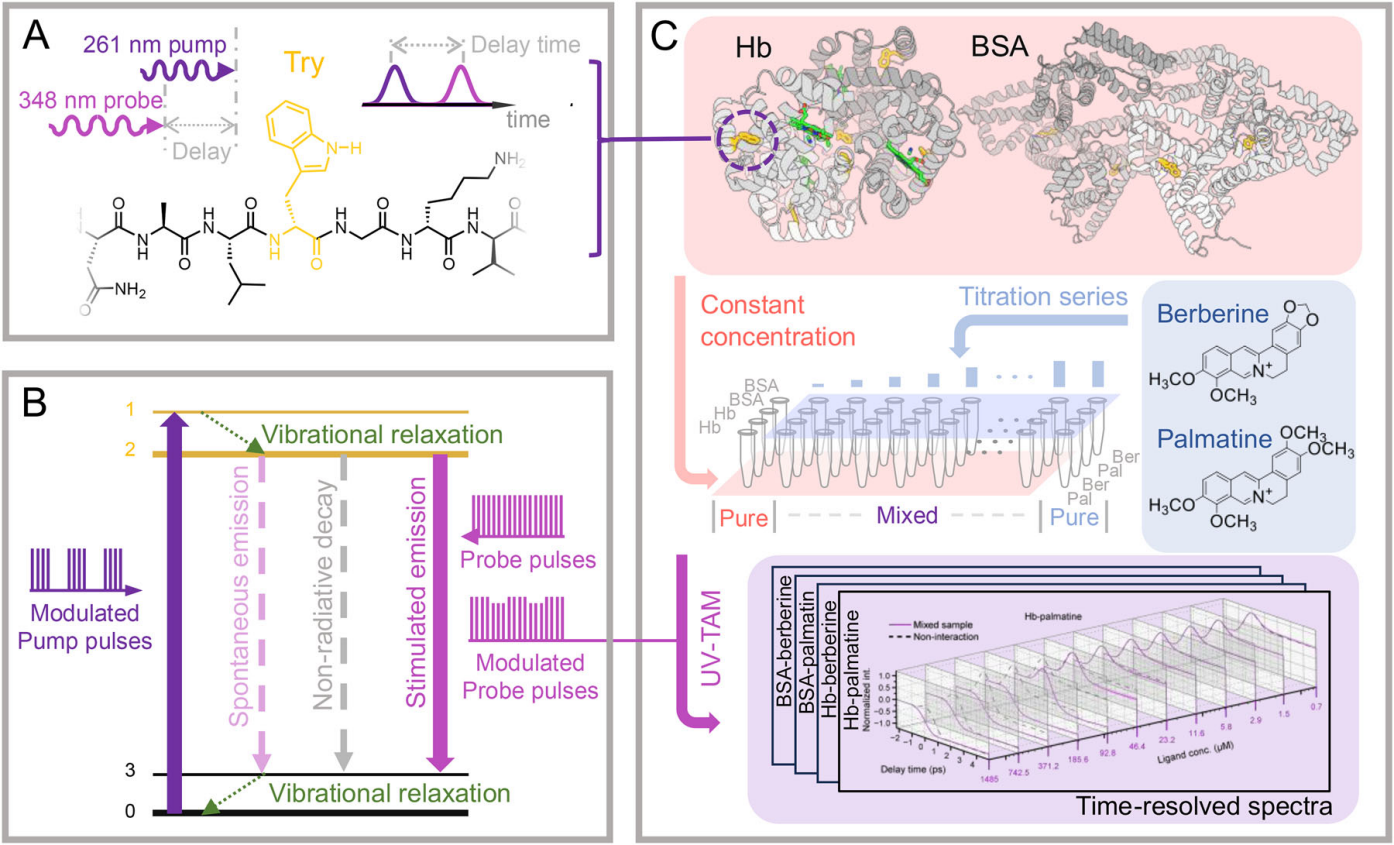
Principle of UV pump‐probe microscopy for protein‐ligand interaction measurement. (A) Schematic illustration of ultraviolet transient absorption microscopy (UV‐TAM) for probing tryptophan residues (shown in gold) in proteins. A femtosecond pump and probe pulse pair arrives at the sample with a controlled time delay to measure the excited‐state dynamics of tryptophan. (B) Energy diagram of a four‐level energy system of tryptophan in a UV‐TAM experiment, in which both spontaneous emission and nonradiative decay can be competed by the stimulated emission with NUV probe stimulation. (C) Workflow of protein–molecule interaction measurement using UV‐TAM. For each protein‐ligand pair, a titration series of the ligand molecule is prepared while maintaining the binding target protein at a constant concentration. Pure protein and ligand solutions are also prepared as controls. For each sample, 1.5 µL of solution is placed between quartz slides and positioned under a reflective UV laser scanning microscope. Pump and probe beams are focused onto the sample to induce transient absorption signals, which are detected using a resonance‐enhanced photodiode and demodulated via a lock‐in amplifier.

Conventional TA techniques generally employ visible or near‐infrared pump and probe wavelengths, which are compatible with common optical components and minimize sample damage [12]. However, different chromophores require distinct pump/probe configurations, complicating the experimental setup and introducing uncertainties for protein detection in binding measurements. To address this, we targeted tryptophan—a common and UV‐active chromophore in proteins—as the reporter. The schematic illustration is shown in Figure 1A. Specifically, we generated the fourth harmonic (261 nm) and third harmonic (348 nm) of a 1045 nm femtosecond laser to serve as the pump and probe beams, respectively, matching tryptophan's absorption and emission characteristics. Figure 1B shows the energy diagram of spontaneous emission, non‐radiative decay, and stimulated emission processes for a UV‐TA experiment. The stimulated emission becomes the dominating decay pathway within the stimulation field of probe pulses. Thus, our method enables highly sensitive, label‐free detection of proteins in solution.

For protein‐ligand binding measurements, a series of UV‐TA experiments was conducted as illustrated in Figure 1C. By maintaining constant protein concentrations and titrating ligand concentrations, time‐resolved transient absorption spectra of both pure and mixed samples were acquired. Ligand binding triggers structural and electronic changes in the protein, including conformational changes, altered rigidity, or charge transfer [12, 14, 15]. These alterations are directly reflected in the excited‐state dynamics captured by UV‐TA spectroscopy. These binding‐induced spectral contrasts, when tracked as a function of ligand concentration, provide a direct and quantitative readout of protein–ligand interactions.

### 2.2 Experimental Setup of UV‐TAM

**FIGURE 2:**
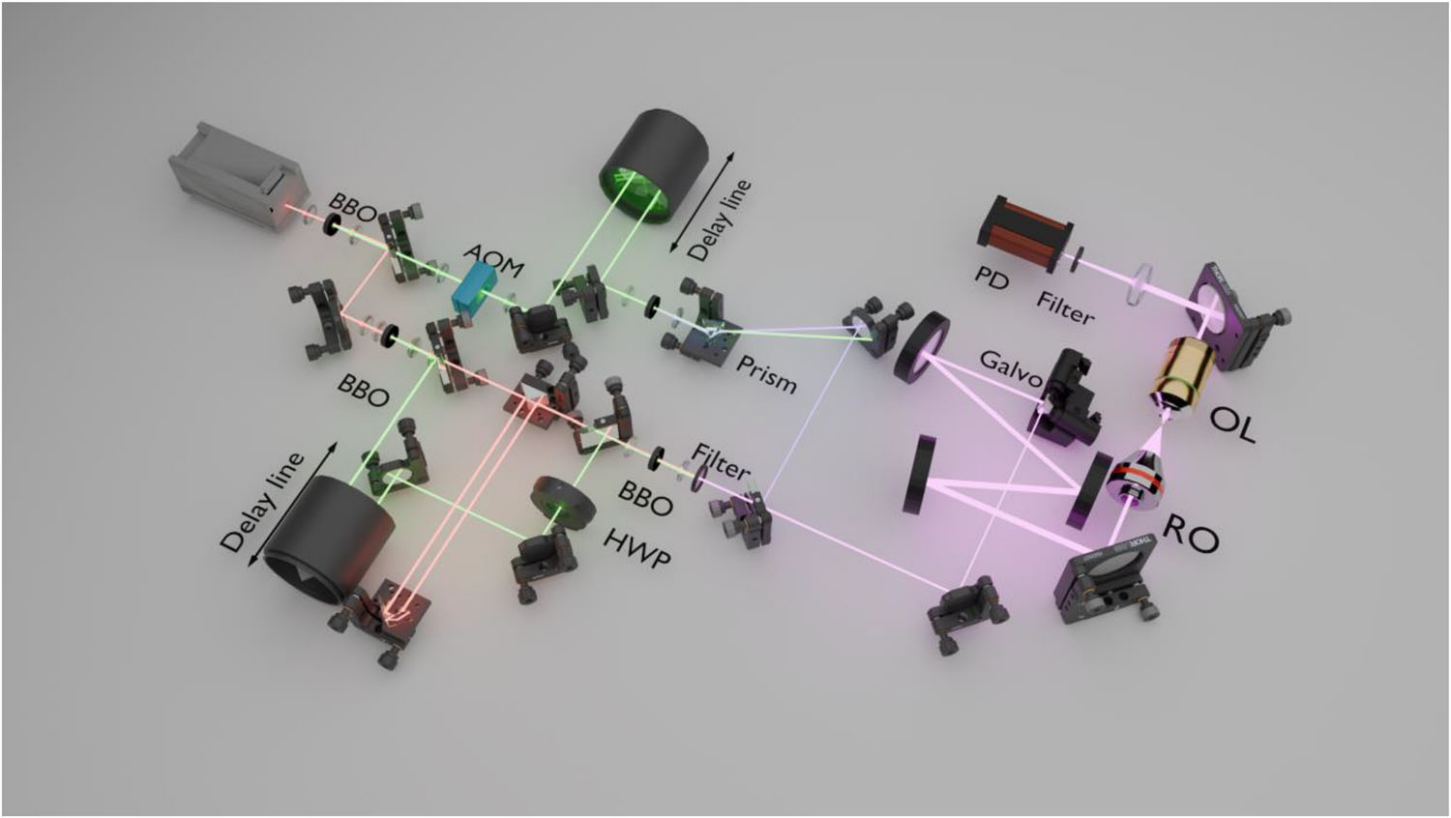
Schematic illustration of the experimental setup of UV‐TAM. BBO: β‐BaB_2_O_4_; DM: dichroic mirror; AOM: acousto‐optic modulator; HWP: half‐wave plate; Galvo: galvo scanning system; RO: reflective objective; OL: objective lens; PD: photo detector.

UV‐TAM directly interrogates proteins in solution by capturing the femtosecond‐resolved pump–probe transient absorption signals at 261 and 348 nm (Figure 2). Briefly, a 1045 nm femtosecond laser was split into two pathways. In the pump arm, 523 nm pulses generated by the first frequency doubling were modulated at 2.68 MHz (AOM) and delayed with a motorized stage before being the second time frequency‐doubled to 261 nm pulses. In the probe arm, the fundamental 1045 nm pulses were tripled to 348 nm pulses. Pump and probe lasers were collinearly combined by a dichroic mirror and delivered to the sample through an all‐reflective, UV‐optimized microscope.

Unlike conventional transient absorption systems, the design of a UV pump‐probe scanning system faces unique difficulties. First, achieving high‐quality of transient absorption measurement is challenging due to severe chromatic aberrations inherent in common transmissive optical components, such as lenses and objectives, in the UV wavelength range, and the prohibitively high cost of UV achromatic lenses or objectives. To overcome this, we employed an all‐reflective module, comprising a galvo scanner, a 4f beam expander with two concave aluminum mirrors, and a 15× reflective objective. This design ensures that the pump and probe beams remain overlapped at the focal point, a prerequisite for sensitive transient absorption measurements, without any chromatic distortion. Also, this reflective design is cost‐effective to facilitate wide adoption in the future. Second, the system minimizes the number of optical components in the UV pathway, as standard materials for lenses and other elements often have high absorbance and poor efficiency in the deep‐UV range. By strategically placing key components like the motorized delay stage and AOM in the visible spectral range before UV generation, the system maintains high efficiency and signal‐to‐noise ratio.

After the sample, the transmitted light was collected using a homemade UV objective lens (composed of calcium fluoride and UV‐grade fused silica lenses). The pump beam was then spectrally filtered out, while the probe beam was detected with a resonance‐tuned photodiode. Amplitude and phase were extracted with a digital lock‐in amplifier. With all the unique designs above, we achieved a sensitivity of Δ*I*/I ≈ 10^−^^7^ at effective focal powers of 150 µW (pump) and 530 µW (probe), which were low enough to prevent UV damage.

For system characterization, concentration‐gradient measurements were performed (Figure S1). Figure S1A presents concentration‐normalized transient absorption spectra for BSA, Hb, berberine, and palmatine. The comparable signal intensities for BSA and Hb—exhibiting high and low fluorescence quantum yields, respectively—confirm that the system's sensitivity is independent of protein fluorescence yield. Figure S1B displays time‐resolved spectra of serial dilutions of BSA and distilled water, while Figure S1C,D show the corresponding phasor analysis. The water response defines the temporal resolution of 0.91 ± 0.01 ps, whereas the limit of detection (LOD) of BSA is below 360 nM, corresponding to fewer than 100 molecules within the focal volume. Detailed procedures for system characterization are provided in the Methods.

### 2.3 Excited‐State Dynamic Alterations Upon Protein Binding

Natural isoquinoline alkaloids are a class of widely sourced, structurally diverse organic compounds with various biological activities, including anticancer, antibacterial, and anti‐inflammatory effects. Among them, berberine and palmatine are classic subjects of research and have gained significant attention in anti‐cancer drug development. However, to explore their potential as therapeutic agents, further studies are needed to investigate their binding affinity with functional proteins and their transport mechanisms in the circulatory system [16]. For instance, stronger binding between drugs and plasma albumin reduces bioavailability while prolonging half‐life [17]. Two plasma proteins— BSA and Hb—were selected as target binding proteins, with berberine and palmatine as ligands, to validate UV‐TAM for measuring protein‐ligand interactions. These two proteins play crucial roles in blood transport and can bind to berberine and palmatine, which are two representative bioactive small‐molecule drugs.

**FIGURE 3:**
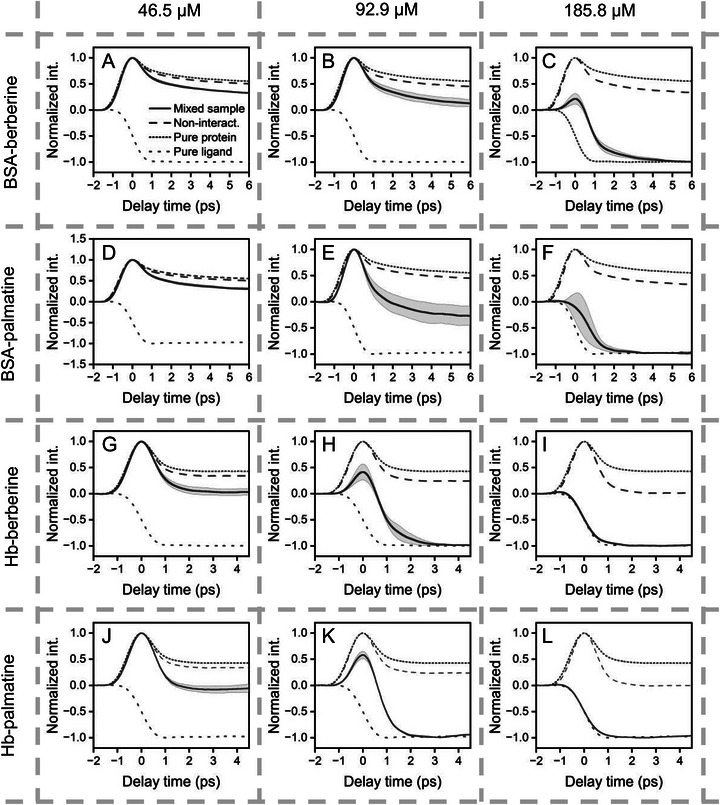
Normalized time‐resolved spectra of protein‐ligand mixture solutions. The ligand concentration was titrated from 46.5 to 185.8 µM for each protein‐ligand pair: (A‐C) BSA and berberine; (D‐F), BSA and palmatine; (G‐I), Hb and berberine; (J‐L), Hb and palmatine. All panels share the same legend shown only in (A): measured spectra are solid lines (shaded areas indicate ±1 SD, n = 3); spectra of pure proteins and ligands are shown as closely spaced dotted lines and widely spaced dotted lines, respectively; non‐interaction spectra calculated from pure samples are plotted as dashed lines.

The workflow of measuring protein‐ligand interactions using UV‐TAM is illustrated in Figure 1C. For the mixed solutions, the constant concentrations of BSA and Hb were set at 147 and 73 µM, respectively, while the ligand was titrated from 700 to 1.49 mM. For each sample, 1.5 µL of solution was placed between quartz slides and positioned under the reflective UV laser scanning microscope. Detailed sample preparation and experimental workflow are provided in the Methods. The transient absorption time‐resolved mean spectra (n = 3, with standard deviation plotted as shaded region) of pure and mixed solutions are plotted in Figure S1A (unit‐concentration time‐resolved spectra) and Figure S2 (max‐min normalized time‐resolved spectra), respectively. Figure 3 presents the normalized transient absorption time‐resolved spectra of mixed solutions where the ligand concentration approaches the protein concentration (46.5–185.8 µM ligand concentrations). UV‐TAM signals display positive and negative (i.e., bipolar) multi‐exponential dynamics resulting from a broad range of physical mechanisms: excited state absorption (ESA) produces negative signals while ground‐state depletion (GSD) and stimulated emission (SE) produce positive signals [12].

Comparing the experimentally measured spectra with the non‐interaction spectra—calculated using the unit‐concentration time‐resolved spectra of the pure samples, plotted as dashed lines in Figures S2 and S3— reveals clear signatures of protein‐alkaloid binding. In Figure 3, normalized spectra of pure samples are also plotted as dotted lines for comparison, with proteins represented by closely spaced (double‐dotted) lines and ligands by widely spaced lines. In the BSA–berberine system (Figure 3A–C), increased berberine concentration leads to a polarity change (from positive signals to bipolar signals) in the transient absorption signal and a pronounced decrease in BSA's excited‐state lifetime, which illustrates a strong interaction between BSA and berberine. This lifetime reduction undermines the spontaneous emission. It is consistent with fluorescence quenching observed upon berberine binding to BSA's Sudlow site I [16]. The other three cases—BSA–palmatine (Figure 3D–F), Hb–berberine (Figure 3F–H), and Hb–palmatine (Figure 3I–L)—show strong changes in time‐resolved spectra, indicating stronger interactions. Notably, in Figure 3C,F,I,L, the negative photothermal signal dominates, which is likely due to enhanced photothermal conversion within the protein–ligand complexes. This enhancement originates from the increased non‐radiative decay pathways upon binding, which accelerate excited‐state decay and promote heat release. The resulting photothermal response serves as an additional signature of binding, complementary to the lifetime shortening observed in the excited‐state absorption.

### 2.4 Binding Affinity Quantification Based on Time‐Resolved Spectra

The excited‐state dynamics of mixed samples and pure samples enable the measurement of protein‐ligand interactions. To further demonstrate the quantitative analytical capability of UV‐TAM, we calculated the *K*_D_ of Hb with two ligands.

Time‐domain analysis of multi‐component systems (free proteins, ligands, and protein‐ligand complexes) encounters practical challenges in maintaining stable signal intensity and signal‐to‐noise ratio (SNR) during prolonged titration measurements. To overcome these limitations while maintaining experimental simplicity, we employed the phasor analysis to analyze normalized time‐resolved decay curves. Phasor analysis is independent of absolute signal intensity and does not require any initial kinetic assumptions [10]. It has become a mature and widely used tool for quantitative component analysis in fluorescence lifetime imaging microscopy (FLIM) [18], and has been successfully extended to nonlinear pump–probe microscopy [15].

In the phasor analysis, time‐resolved signals are decomposed into Fourier components *g* and *s*, representing the real and imaginary parts, respectively. Mathematically, the phasor transformation projects kinetic profiles onto coordinates in the phasor domain:

(1) $${\vec{P}}_{\omega }\left(I\left(t\right)\right)=\left(\frac{\langle I\left(t\right),\;\cos\left(\omega t\right)\rangle }{∥I\left(t\right){∥}_{1}},\frac{\langle I\left(t\right),\;\sin\left(\omega t\right)\rangle }{∥I\left(t\right){∥}_{1}}\right)=\left(g,s\right)$$

where *I*(*t*) represents the time‐resolved signal, and *ω* is an optimized frequency parameter. $∥I\left(t\right){∥}_{1}=\int \left|I(t)\right|dt$ represents the *L*_1_‐norm of the signal intensity.

The phasor distance metric:

(2) $$L_{\mathrm{phasor}}={\left\|{\vec{P}}_{\omega }\left(I_{\mathrm{interaction}}\left(t\right)\right)\;-\;{\vec{P}}_{\omega }\left(I_{\mathrm{non}-\mathrm{interaction}}\left(t\right)\right)\right\|}_{2}$$

quantifies the binding‐induced changes via Euclidean separation between: (i) ${\vec{P}}_{\omega }\left(I_{\mathrm{interaction}}\left(t\right)\right)$: experimental phasor coordinates of interacting mixed samples. (ii) ${\vec{P}}_{\omega }\left(I_{\mathrm{non}-\mathrm{interaction}}\left(t\right)\right)$: theoretical non‐interaction phasor coordinates derived from pure component spectra.

Our derivation reveals an approximately linear relationship expressed as:

(3) $$f_{\mathrm{bound}}\approx \kappa \cdot L_{\mathrm{phasor}}\cdot ∥I_{\mathrm{non}-\mathrm{interaction}}\left(t\right){∥}_{1}$$

where *f*_bound_ represents the bound protein fraction, and κ represents the empirically determined scaling factor. The *K*_D_ values were determined by fitting the binding fractions to the law of mass action (details are provided in Methods).

To quantify the dissociation constant for Hb‐berberine binding, we optimized the frequency *ω* through dual criteria: (i) adequate phasor‐domain separation between Hb and berberine to resolve the binding‐induced changes above noise floor, and (ii) selection of a relatively high frequency within the operational range to suppress the photothermal background interference, because photothermal contributions arise from slow thermal diffusion processes that dominate the low‐frequency phasor domain. Although the enhancement of the photothermal component serves as a qualitative signature of protein–ligand binding, it does not provide quantitative information about excited‐state population changes and introduces substantial variability when used for equilibrium analysis. This variability is evident in the time‐resolved spectra of Figure 3, where the standard‐deviation shading broadens significantly at longer delay times, reflecting the increased fluctuation of the photothermal component.

**FIGURE 4:**
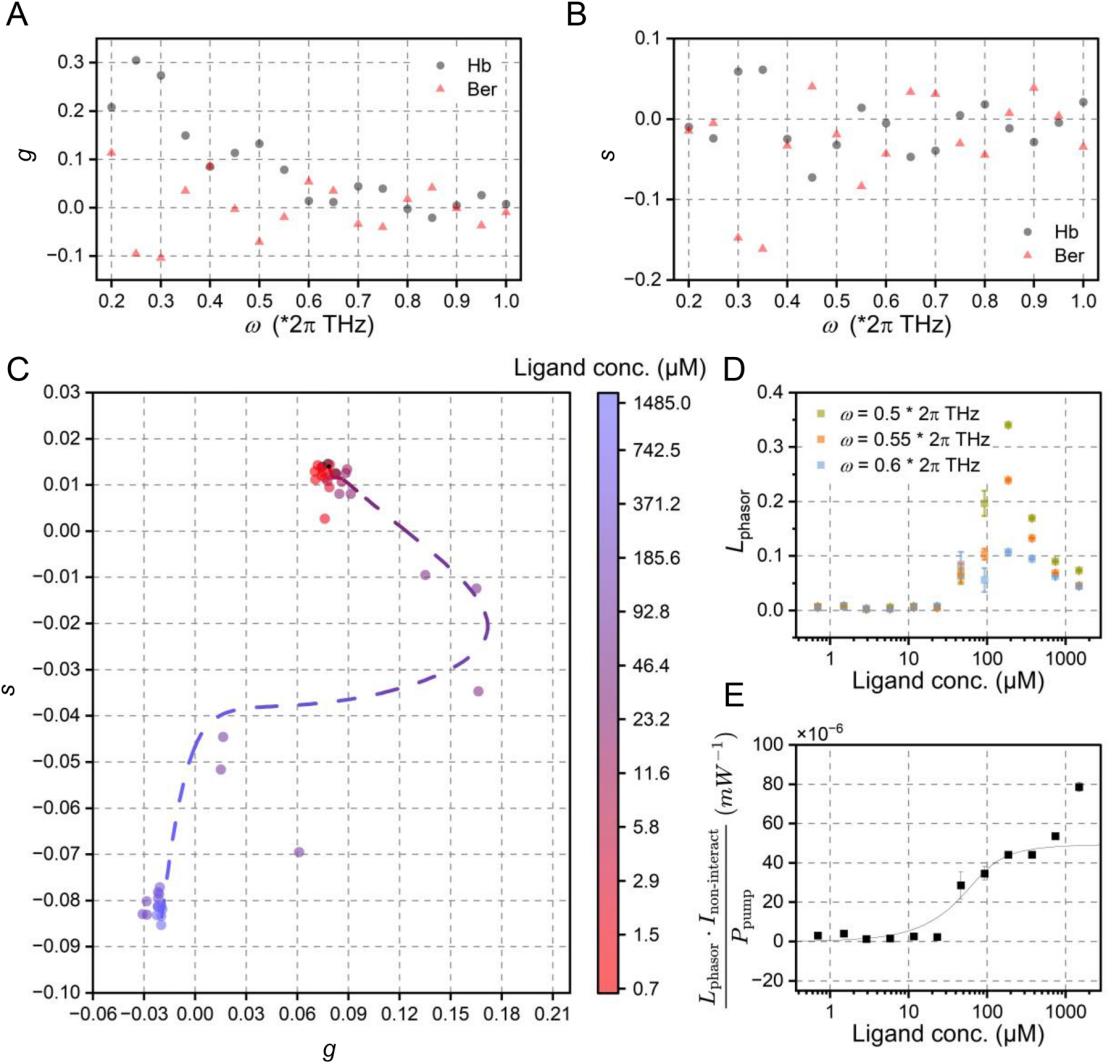
Phasor analysis‐based estimation of the dissociation constant for Hb‐berberine binding. (A) Phasor component *g* of the time‐resolved spectra for pure Hb and berberine. (B) Phasor components of the time‐resolved spectra for pure Hb and berberine. (C) Scatter phasor plot of pure samples and titration gradient mixtures at a frequency of 0.55 × 2π THz, with a dashed line indicating the corresponding phasors assuming no protein‐ligand interaction. (D) Phasor distances between mixed samples with and without protein‐ligand interaction in the phasor domain at frequencies near 0.55 × 2π THz. (E) The binding curve was approximated from phasor distance, showing a binding affinity of (15.5 ± 6.7) µM.

As shown in Figure 4A,B, we systematically evaluated *g* and *s* components of Hb and berberine at frequencies across 0.2–1.0 × 2π THz, identifying 0.55 × 2π THz as the optimal frequency where both components showed the most distinct separation. Figure 4C displays the phasor plot of pure samples and titration series (0.7 ‐ 1486.5 µM ligand concentration), with connecting lines tracing the theoretical non‐interaction trajectory ordered by ligand concentration [L_0_]:

(4) $${\left\{{\vec{P}}_{\omega }\left(I_{\mathrm{non}-\mathrm{interaction}}\left(t\right)\right)\right\}}_{\left[L_{0}\right]=0.7\;\mu M}^{1486.5\;\mu M}$$

Figure 4D shows corresponding phasor distances in the phasor domain at frequencies near 0.55 × 2π THz, where minimal error dispersion (standard deviation, n = 3) at this optimal frequency demonstrates the critical importance of frequency selection. Phasor distances across varying [L_0_] showed maximum deviations at 46.4 and 92.8 µM, corresponding to bipolar signals (Figure 3G,H). At these points, polarity switching introduced vectorial ambiguity in phasor analysis, increasing computational uncertainty. Binding‐induced changes increased progressively and reached a maximum at 185.6 µM, beyond which signal dilution attenuated the response. Correction using ‖*I*_non − interaction_(*t*)‖_1_ yielded the near‐saturation curve in Figure 4E, showing a binding affinity of (15.5 ± 6.7) µM. Significant deviation at maximal [L_0_] (1486.5 µM) likely results from error amplification during the intensity‐based correction: non‐converging phasor distances cause the product term to diverge asymptotically instead of approaching the baseline.

**FIGURE 5:**
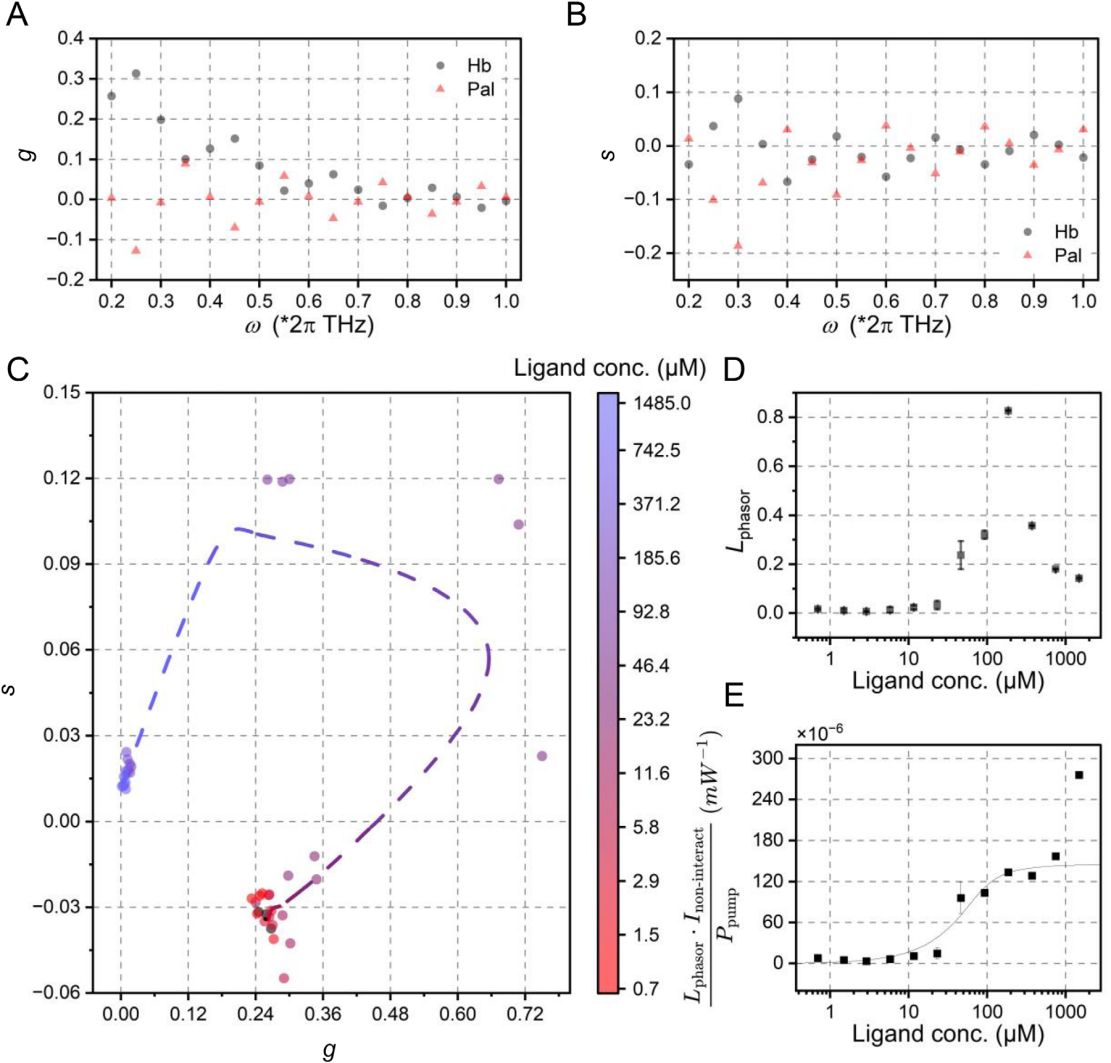
Phasor analysis‐based estimation of the dissociation constant for Hb‐Palmatine binding. (A) Phasor component *g* of the time‐resolved spectra for pure Hb and palmatine. (B) Phasor components of the time‐resolved spectra for pure Hb and palmatine. (C) Scatter phasor plot of pure samples and titration gradient mixtures at a frequency of 0.25 × 2π THz, with a dashed line indicating the corresponding phasors assuming no protein‐ligand interaction. (D) Phasor distances between mixed samples with and without protein‐ligand interaction in the phasor domain at a frequency of 0.25 × 2π THz. (E), The binding curve was approximated from phasor distance, showing a binding affinity of (11.2 ± 6) µM.

The workflow for determining the Hb‐palmatine dissociation constant followed the same methodology. As shown in Figure 5A,B, palmatine's phasor components exhibited a limited separation from Hb at higher frequencies. We therefore selected 0.25 × 2π THz as the optimized frequency, where maximal phasor discrimination was achieved. Figure 5C displays the corresponding phasor plot. Phasor distances at this frequency (Figure 5D) were used to generate the binding curve (Figure 5E), yielding a dissociation constant of 11.2 ± 6 µM. Notably, the dissociation constants calculated from our analysis closely match those obtained via ITC measurements reported in the prior literature [11], which showed 15.4 µM for berberine and 11.5 µM for palmatine.

## 3 Conclusions

In this work, we developed a label‐free, immobilization‐free methodology—UV‐TAM —to address critical limitations in measuring protein‐ligand interactions under physiological conditions. Conventional binding assays often require fluorescent or radioactive labeling or surface immobilization, which may perturb the native binding process: labels can alter protein conformation or introduce steric hindrance, while immobilization on surfaces may restrict molecular orientation or reduce binding freedom [19]. In addition, labeling and coupling reactions typically involve multiple chemical steps and purification, which are time‐consuming and difficult to optimize for low‐yield or unstable protein preparations such as membrane receptors [6]. In contrast, UV‐TAM is entirely label‐ and immobilization‐free. It directly probes the intrinsic excited‐state dynamics of tryptophan residues in solution under native conditions, thereby avoiding these confounding factors.

Beyond being label‐free, UV‐TAM offers additional features compared with existing label‐free methods. Some of these methods are variations of fluorescence labeling techniques (detecting auto‐fluoresce rather than fluorescent label), such as Label‐free MST [6] and Label‐free DSF [20], but they are limited by the quantum yield of the protein's native fluorescence, which is influenced by the protein's non‐radiative decay. Other methods generally rely on auto‐fluoresce or detecting macroscopic changes in solution properties (ITC measures bulk enthalpy changes and BSI detects binding through refractive index shifts), which can be minimal and hard to detect for certain binding events. For instance, as reported for frHMGB1–TL and CXCL12, where no measurable heat release prevented affinity quantification, ITC becomes ineffective [21]. By contrast, UV‐TAM detects the binding‐induced alterations in femtosecond excited‐state decay kinetics. This signal arises from universal microscopic contrasts—such as conformational changes, rigidity changes, or charge transfer [12, 14, 15]—that occur when a ligand binds to a protein's tryptophan residue. Therefore, UV‐TAM can be a more universally applicable method, especially for interactions where macroscopic changes in heat, refractive index, or charge are too subtle to measure. This microscopic signal is also largely unaffected by non‐radiative decay on a femtosecond timescale, which makes it effective for non‐ or weak‐fluorescent proteins.

To gain a more comprehensive understanding of the positioning and characteristics of UV‐TAM, we compared UV‐TAM to several commonly used molecular interaction detection methods according to previous studies [20, 22], summarizing key metrics in Table 1. Each technique has distinct advantages and limitations. UV‐TAM provides unique insights into excited‐state dynamics, thus simplifying the experimental workflow of UV‐TAM: (i) it requires no labeling or surface immobilization, avoiding sample preprocessing; and (ii) it relies on normalized kinetic profiles rather than absolute signal levels, thereby obviating complex calibration and reference channels. Although it requires no labeling or surface immobilization, UV‐TAM requires low sample volumes (1.5 µL due to the current sample‐loading format), similar to MST, making this method suitable for precious or low‐yield proteins. As mentioned previously, compared with other label‐free methods, UV‐TAM has no limitations from fluorescent efficiency or the magnitude of physical changes during the reaction.

**TABLE 1:** Comparison between UV‐TAM and previously reported experimental affinity determination methods.

Name	Info gained in addition to KD	Required amount of sample	Throughput	Equipment cost	Limitaion
Surface plasmon resonance (SPR)	*k*_on_, *k*_off_	Low‐Medium	Low (1∼2 per day) for kinetic analysis	High	Immobilized protein/ligand. Complex system optimization.
Bio‐layer interferometry (BLI)	*k*_on_, *k*_off_	Low	Medium (< 3 h per experiment)	High	Immobilized protein/ligand. Limited application for tight binders and some molecules.
Differential scanning fluorimetry (DSF)	*T*m	Low	High (50/h for kinetic analysis)	Low	Dependent on a dye or limited by auto‐fluorescence. Many false positives and negatives.
Microscale thermophoresis (MST)	—	Low	Medium (> 2 h per experiment, 96‐well plates available)	Medium	Dependent on a dye or limited by auto‐fluorescence.
Isothermal titration calorimetry (ITC)	Δ*G*_b_, Δ*H*_b_, Δ*S*_b_, stoichiometry	High	Low (∼8 per a 8 h day)	Low	Limited to reaction with detectable heat.
Back‐scattering Interferometry (BSI)	*k*_on_, *k*_off_	Low (Minimum to 350 pL)	N/A	N/A	Limited to proteins displaying detectable changes in the refractive index
Kinetic Capillary Electrophoresis with Mass‐spectrometry Detection (KCE‐MS)	Stoichiometry; *k*_on_, *k*_off_ in certain variants	Low	Low (< 1 h per run), though 96‐well systems are available	High	Limited to binding partners that are separable by electrophoresis.
**Ultraviolet transient absorption microscopy (UV‐TAM)**	**Excited‐state dynamics**	**Low (1.5 µL); potentially reducible to nL scale with optimized loading**	**Low (> 4 h per experiment); scalable via SNR optimization and multiplexed scanning**	**Medium**	**Trade off between thoughput and SNR for prevention of UV damage**.

UV‐TAM has the potential to quantify stronger binding affinities. Methods that determine affinity by the fraction of the bound complex in equilibrium solutions are fundamentally constrained by their concentration limit of detection, which sets the minimum *K*_D_ that can be reliably extracted. For example, the quantitative capability of the enthalpy‐array calorimetry was restricted by its LOD of 50 µM [23, 24]. In the current UV‐TAM setup, only µM‐scale *K*_D_ values can be reliably determined because the LOD is approximately 0.36 µM, as detailed in the Methods section. This range is insufficient for certain drug discovery applications, such as protein–aptamer bindings with *K*_D_ values in the nM to pM range (e.g., thrombin [25, 26], TNF‐alpha [27], VEGF [28], and Hb [29]). Additional experiments regarding protein–aptamer interactions are provided in the Supporting Information (Figure S3). This limitation is not intrinsic to the UV‐TAM method but arises from engineering constraints of the current prototype: (i) the UV optical power of sub‐mW levels to avoid photodamage, which is lower than the mW‐level excitation commonly used in MST [30] and label‐free MST [6]. (ii) the signal generation confined to a tiny focal volume of only 0.44 fL, where even a single molecule corresponds to an effective concentration of 3.8 nM. Therefore, increasing the focal volume into the pL–nL range offers a practical route to enhance sensitivity: if proportionally scaling the optical power to maintain peak irradiance for TA detection, the volume of interrogated solution would increase by several orders of magnitude, thereby reducing the LOD by a comparable extent (10^3^–10^6^), enabling future UV‐TAM systems to cover a broader dynamic range of *K*_D_ values.

UV‐TAM also holds promise for supporting smaller sample volumes and achieving higher throughput. The current sample consumption is mainly constrained by the loading approach because the UV‐TAM signal originates predominantly from the focal‐plane interrogation volume, which is on the order of one nanoliter, and additional sample volume does not contribute to the measurement. Similar to many laser‐scanning microscopies, UV‐TAM can therefore be adapted to capillaries or microfluidic platforms that require minimal sample amounts. Moreover, integrating UV‐TAM with microfluidics enables automated chip‐based assay formats, which not only improve SNR by minimizing optical path length and background scattering but also potentially support high‐throughput analysis.

## 4 Methods

### 4.1 Instrumentation

The setup of UV‐TAM is shown in Figure 2. The system was constructed around a Spectra‐Physics HighQ‐2 femtosecond laser (1045 nm, 200 ± 50 fs, 63 MHz) operating at 1.8 W output power. The fundamental 1045 nm infrared beam was initially frequency‐doubled using a β‐BaB_2_O_4_ (BBO) crystal (23.1° cut angle, 1.1 mm thick) to produce the visible beam at 523 nm, which was separated from residual infrared via a 650 nm short‐pass dichroic mirror. For pump beam generation, the 523 nm beam underwent intensity modulation by an acousto‐optic modulator (AOM, 2.68 MHz) and precise interpulse temporal delay control via a motorized delay line (LTS300C/M, Thorlabs), followed by another second harmonic generation in a BBO crystal (1 mm thick) to yield 261 nm beam. The residual visible beam was filtered using a CaF_2_ prism exploiting refractive index dispersion. The probe beam at 348 nm was generated by tripling the 1045 nm fundamental beam through a cascaded nonlinear process: initial frequency doubling to 523 nm, followed by sum‐frequency mixing of the residual 1045 nm and the generated 523 nm beams in a BBO crystal (I‐type phase matching). The pump and probe beams were collinearly combined using a dichroic mirror (FF310‐Di01‐25x36, Semrock).

Critical to UV performance was a homemade all‐reflective microscope. A galvanometer scanner (SS8107CM, Sunny) coupled to a reflective 4f beam expander (150/500 mm aluminum concave mirrors) enabled raster scanning while avoiding chromatic aberrations. Overlapped beams were focused onto samples via a reflective objective (LMM15X‐UVV, Thorlabs). Post‐sample probe beam was collected by a homemade CaF_2_/fused silica objective and filtered (FBH351‐10 bandpass, Thorlabs).

To detect the subtle UV pump‐probe signal, we employed a two‐stage noise suppression strategy: First, a resonance‐amplified photodiode with high‐Q LC circuit (center frequency 2.5 MHz, gain 24.8 dB) pre‐amplified modulated signals. Second, subsequent processing by a digital lock‐in amplifier (LIA, MFLI 5 MHz, Zurich Instruments) extracted the amplitude and phasor components with sub‐nV sensitivity. This enabled detection of modulation depths down to Δ*I*/*I* ∼10^−^^7^ while preventing UV‐induced sample damage (Average powers at sample: 150 µW pump, 530 µW probe; corresponding peak power densities: 63 kW/cm^2^ pump, 120 kW/cm^2^ probe).

### 4.2 Sample Preparation

Protein stock solutions were prepared by dissolving BSA (56.4 mg in 1.2 mL) or Hb (8.5 mg in 0.6 mL) in sterile water, followed by centrifugation at 8,000 rpm for 3 min, to obtain concentrations of 707 and 147 µM, respectively. The BSA stock was threefold diluted to 236 µM for binding studies. Alkaloid stock solutions (berberine and palmatine, 2.97 mM) were similarly prepared in sterile water and centrifuged. For binding studies, alkaloid solutions underwent serial dilution across a concentration range of 1.5 to 2.97 mM. Equal volumes of diluted alkaloid solutions and fixed concentration protein solutions (236 µM BSA or 147 µM Hb) were mixed and equilibrated at ambient temperature for 24 h. Samples (1.5 µL from both pure and mixed solutions) were loaded between UV‐transmissive fused silica windows (20 × 20 × 0.5/0.25 mm^3^), forming a microcuvette with a path length of < 0.12 mm.

### 4.3 Experimental Workflow

To minimize potential photodamage to proteins (manifested as protein precipitation or fluorescence quenching of BSA) arising from prolonged exposure despite low average laser power at the sample, a scanning and integration strategy was adopted in lieu of single‐point acquisition. Each solution sample under the objective lens was sandwiched between two quartz slides (0.25 mm and 0.5 mm thick), with the thinner quartz slide facing the objective lens. During experiments, the galvanometer kept scanning 100 pixels in both the *X*‐axis and *Y*‐axis with a dwell time of 10 µs per pixel, yielding a field of view of 85 µm × 170 µm. Concurrently, the time constant of LIA was set to 1 s. After the time delay was set to 0, the LIA's reference phase was meticulously adjusted to maximize the X‐channel output of LIA. Following this, the sample position along the Z‐axis was optimized to achieve peak signal intensity. Signal acquisition commenced as the optical path difference between pump and probe was scanned from −0.5 mm to a final position of +1.5 mm for samples containing BSA or +1.0 mm for samples containing Hb. All pure solution samples—BSA, Hb, berberine, and palmatine—were tested first, followed by measurements on the mixed solution samples.

### 4.4 System Characterization

As shown in Figure S1a, the signal intensities of BSA and Hb detected by our system were essentially comparable. Therefore, BSA was used to test the system's detection limit for Hb. Figure S1b displays the time‐resolved spectra of BSA at concentrations ranging from 180 nM to 2.9 µM. At extremely low protein concentrations, the protein signal no longer dominates, and the pump‐probe time‐resolved spectra of the solution gradually approach a weak, nearly Gaussian fixed signal originating from THz excitation in resonance with the rotational damping motion of water molecules [31]. The water response, approximated as the instrument response function, yields a temporal resolution of 0.91 ± 0.01 ps from the Gaussian‐fitted FWHM.

Phasor analysis was employed to calculate the phasor components of the time‐resolved spectra at various THz angular frequencies, with the results presented in Figure S1c,d. For components at most frequencies, 0.36 µM BSA could be clearly distinguished from 0.73 µM BSA, with the most significant separation observed at 0.30 × 2π THz (two‐tailed independent samples t‐test, t = −3.73, p = 0.02, n = 3). The phasor components of 0.18 µM BSA solution and distilled water showed significant separation only at specific angular frequencies (e.g., 0.75 × 2π THz) and were not statistically significant at the 0.05 level.

Based on these findings, the detection limit of this transient absorption system for protein concentration is determined to be < 360 nM, corresponding to <100 molecules within the focal volume of the objective lens. This result demonstrates the potential of the UV pump‐probe absorption system for label‐free detection of precious protein samples, achieving sensitivity within two orders of magnitude of single‐molecule detection. The approach shows significant application value for the concentration measurement of valuable proteins.

### 4.5 Dissociation Constant Estimation

To derive the dissociation constant *K*_D_, we identify a concentration‐dependent nonlinear parameter dominated by binding fraction dynamics. The phasor distance—defined as the *L*_2_‐norm separation between experimental and theoretical non‐interaction phasor coordinates in the phasor domain—serves as this key parameter. To simplify the derivation, time‐resolved spectra *I*(t) are represented as infinite‐dimensional vectors $\vec{x}$. The phasor transformation (Equation) becomes:

(5) $${\vec{P}}_{\omega }\left(\vec{x}\right)=\frac{\vec{F_{\omega }}\left(\vec{x}\right)}{{\left\|I\left(t\right)\right\|}_{1}}$$

where Fω⃗=(Re[FT(x⃗)(ω)],Im[FT(x⃗)(ω)]) is the vectorial Fourier transform. For ligand concentration [L_0_], the measured signal is dominated by three components—free binding target (B), free ligand (L), and complex (BL):

(6) $${\vec{x}}_{\left[L_{0}\right]}=\left[B\right]\vec{b}+\left[L\right]\vec{l}+\left[\mathrm{BL}\right]\vec{bl}$$

with [B], [L], [BL] denoting the concentrations and $\vec{b}$, $\vec{l}$, $\vec{bl}$ denoting unit‐concentration spectra. The phasor transformation yields:

(7) $${\vec{P}}_{\omega }\left(\vec{x_{\left[L_{0}\right]}}\right)=\frac{\left[B\right]{\vec{F}}_{\omega }\left(\vec{b}\right)+\left[L\right]{\vec{F}}_{\omega }\left(\vec{l}\right)+\left[\mathrm{BL}\right]{\vec{F}}_{\omega }\left(\vec{bl}\right)}{∥\left[B\right]\vec{b}+\left[L\right]\vec{l}+\left[\mathrm{BL}\right]\vec{bl}{∥}_{1}}$$

Assuming negligible impact of binding on total signal intensity:

(8) $$\begin{array}{ccc} & & |\left[B\right]\vec{b}+\left[L\right]\vec{l}+\left[\mathrm{BL}\right]\vec{bl}{∥}_{1}\\ & & =∥\left[B_{0}\right]\vec{b}+\left[L_{0}\right]\vec{l}+\left[\mathrm{BL}\right]\left(\vec{bl}-\vec{b}-\vec{l}\right){∥}_{1}\approx ∥\vec{x_{0\left[L_{0}\right]}}{∥}_{1} \end{array}$$

where $\vec{x_{0[L_{0}]}}=\left[B_{0}\right]\vec{b}+\left[L_{0}\right]\vec{l}$ is the theoretical non‐interaction spectra calculated from pure solution spectra. The phasor analysis results are:

(9) $${\vec{P}}_{\omega }\left(\vec{x_{\left[L_{0}\right]}}\right)\&\approx \frac{\left[B_{0}\right]{\vec{F}}_{\omega }\left(\vec{b}\right)+\left[L_{0}\right]{\vec{F}}_{\omega }\left(\vec{l}\right)+\left[\mathrm{BL}\right]{\vec{k}}_{\omega }}{∥\vec{x_{0\left[L_{0}\right]}}{∥}_{1}}$$

(10) $${\vec{P}}_{\omega }\left(\vec{x_{0\left[L_{0}\right]}}\right)=\frac{\left[B_{0}\right]{\vec{F}}_{\omega }\left(\vec{b}\right)+\left[L_{0}\right]{\vec{F}}_{\omega }\left(\vec{l}\right)}{∥\vec{x_{0\left[L_{0}\right]}}{∥}_{1}}$$

where ${\vec{k}}_{\omega }={\vec{F}}_{\omega }\left(\vec{bl}\right)-{\vec{F}}_{\omega }\left(\vec{b}\right)-{\vec{F}}_{\omega }\left(\vec{l}\right)$ is the complex vector difference per unit concentration. The difference vector satisfies:

(11) $$\left[{\vec{P}}_{\omega }\left(\vec{x_{\left[L_{0}\right]}}\right)-{\vec{P}}_{\omega }\left(\vec{x_{0\left[L_{0}\right]}}\right)\right]\cdot ∥\vec{x_{0\left[L_{0}\right]}}{∥}_{1}\approx f_{\mathrm{bound}}\cdot {\vec{k}}_{\omega }\cdot \left[B_{0}\right]$$

which can be collapsed to scalar form via *L*_2_‐norm:

(12) $$f_{\mathrm{bound}}\approx \frac{1}{∥{\vec{k}}_{\omega }{∥}_{2}\cdot \left[B_{0}\right]}\cdot ∥{\vec{P}}_{\omega }\left(\vec{x_{\left[L_{0}\right]}}\right)-{\vec{P}}_{\omega }\left(\vec{x_{0\left[L_{0}\right]}}\right){∥}_{2}\cdot ∥\vec{x_{0\left[L_{0}\right]}}{∥}_{1}$$

This establishes the linear relationship required for saturation curve fitting (Equation). Applying the law of mass action:

(13) $$\begin{array}{ccc} & & f_{\mathrm{bound}}\\ & = & \frac{\left(\left[L_{0}\right]+\left[B_{0}\right]+K_{D}\right)-\sqrt{{\left(\left[L_{0}\right]+\left[B_{0}\right]+K_{D}\right)}^{2}-4\cdot \left[L_{0}\right]\cdot \left[B_{0}\right]}}{2\left[B_{0}\right]} \end{array}$$

combining Equations 12 and 13 gives Equation 14:

(14) $$\begin{array}{ccc} {\left\|{\vec{k}}_{\omega }\right\|}_{2}\cdot & & \frac{\left(\left[L_{0}\right]+\left[B_{0}\right]+K_{D}\right)-\sqrt{{\left(\left[L_{0}\right]+\left[B_{0}\right]+K_{D}\right)}^{2}-4\cdot \left[L_{0}\right]\cdot \left[B_{0}\right]}}{2}\\ & & \approx L_{\mathrm{phasor}}\cdot {\left\|\vec{x_{0\left[L_{0}\right]}}\right\|}_{1} \end{array}$$

where only *K*_D_ and ${∥{\vec{k}}_{\omega }∥}_{2}$ are fitted parameters.

This approach only requires system stability during the measurement of unit‐concentration pure component spectra $\vec{b}$ and $\vec{l}$ (typically 10–20 min). For mixed samples, it solely relies on preserving the temporal profile of time‐resolved spectra, substantially reducing experimental complexity.

### 4.6 Statistical Analysis

For UV‐TAM time‐resolved spectra, Savitzky–Golay filtering with a time window of 1.54 ps was applied. All normalization procedures were performed using min–max scaling, with specific representations indicated by the y‐axis labels in the corresponding figures. For UV–vis absorption spectra, baseline correction only was applied. Data are presented as mean ± standard deviation (SD) where applicable. Shaded regions and error bars shown in all figures represent the SD calculated from n = 3 independent measurements. Statistical comparisons between two groups were performed using a two‐tailed independent samples Student's t‐test, as indicated in the corresponding figure legends, with a significance level of α = 0.05. Nonlinear fitting for *K*_D_ estimation was performed using the Trust Region Reflective algorithm implemented in Python. All data processing and statistical analyses were carried out using Python and Origin.

## Conflicts of Interest

The authors declare no conflict of interest.

## Supporting information




**Supporting File**: advs74035‐sup‐0001‐SuppMat.docx.

## Data Availability

The data that support the findings of this study are available in the supplementary material of this article.
