# Supplementary material for: Label‐Free and Immobilization‐Free Protein‐Binding Assays by Ultraviolet Transient Absorption Microscopy
Source: Adv Sci (Weinh). 2026 Jan 27;13(19):e19985. doi: 10.1002/advs.202519985 (PMC13045408; doi:10.1002/advs.202519985)
Supplement: Supplementary file 1 — Supporting File: advs74035‐sup‐0001‐SuppMat.docx. [file ADVS-13-e19985-s001.docx]

Supporting Information

Label-free and Immobilization-free protein-binding assays by Ultraviolet Transient Absorption Microscopy

Jianghao Shen, Qiangqiang Wang, Fan Wu, Yang Gao, Lu Lan ^*^, Pu Wang ^*^


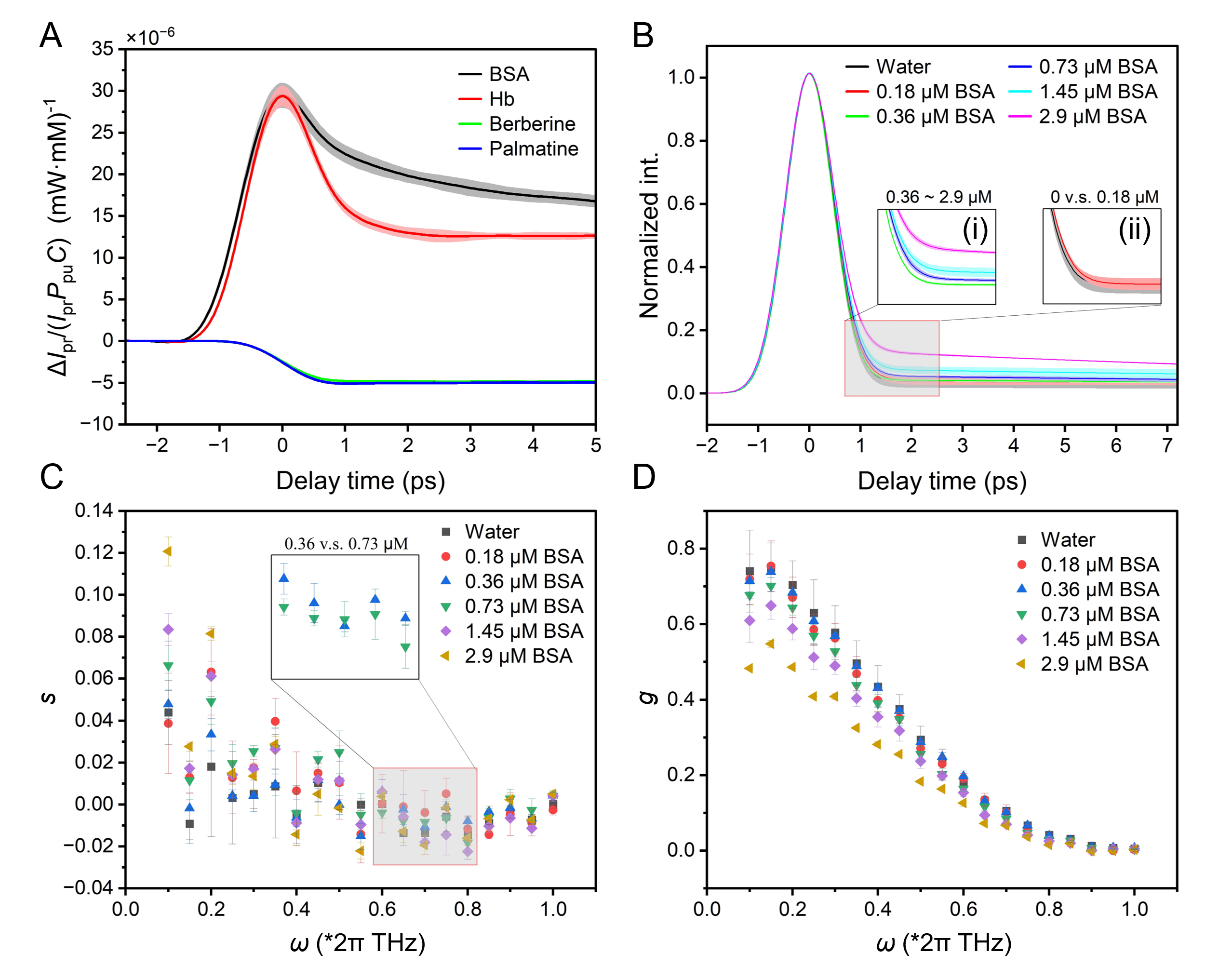


**Figure S1. System characterization of UV-TAM.** (**A**) Time-resolved UV-TA spectra of pure protein and ligand molecule solutions normalized to unit concentration and pump power; (**B**) Time-resolved UV-TA spectra of BSA at concentrations ranging from 0 to 2.9 μM, with zoom-in view from delay time of 0.7 to 2.5 ps highlighting: (i) the spectral region for concentrations 0.36–2.9 μM; (ii) the spectral region for 0.18 μM concentration. (**C**) Phasor component *s* for solutions at each concentration. (Inset: data highlighting the region around 0.7 × 2π THz, contrasting differences between 0.36 μM and 0.73 μM BSA solutions). (**D**) Phasor component *g* for solutions at each concentration.


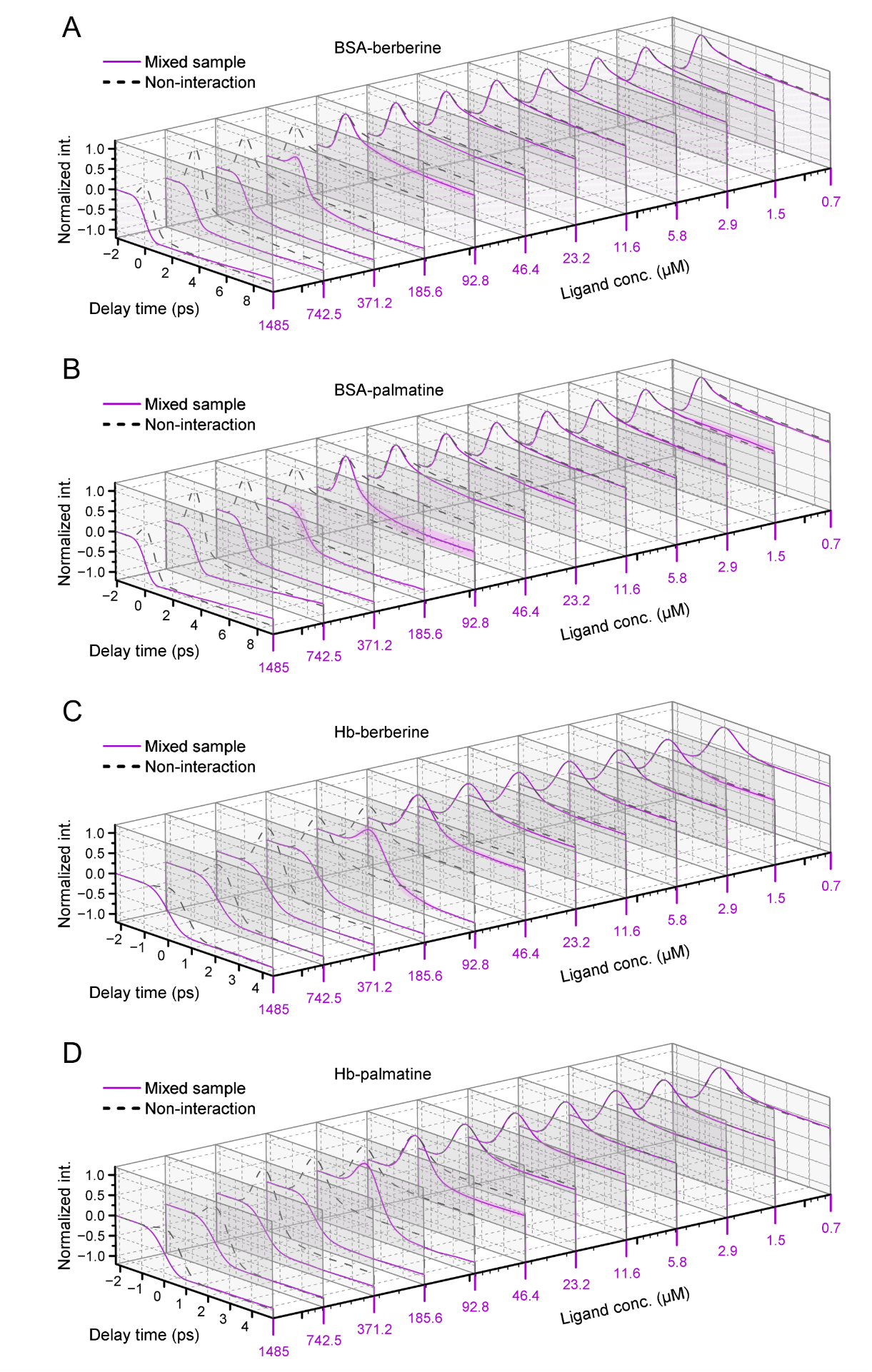


**Figure S2. Normalized time-resolved spectra of protein-ligand mixture solutions.** The ligand molecule was titrated from 700 nM to 1.5 mM for each protein-ligand binding pair mixture: (**A**) BSA and berberine. (**B**) BSA and palmatine. (**C**) Hb and berberine. (**D**) Hb and palmatine. The non-reactive results calculated from pure sample solutions are plotted as dash lines.

**Additional experiments**

DNA aptamers have been widely used as affinity ligands for protein recognition. To further evaluate whether transient absorption (TA) spectroscopy can probe protein–aptamer interactions, additional control experiments were performed using a hemoglobin (Hb)–aptamer system. A previously reported 72-mer DNA sequence capable of binding Hb was selected, while a length-matched DNA sequence reported to bind HbA1c was used as a control sequence.[1]

The interaction was characterized using both UV–Vis absorption and transient absorption spectroscopy. Due to the pronounced nucleobase absorption band near 260 nm, excitation at 260 nm would introduce substantial background signals. Therefore, TA measurements were carried out using a 520 nm pump (2 mW on sample) and a 780 nm probe (10 mW on sample). Due to the current LOD of the UV-TAM system, these experiments were intended for qualitative comparison rather than quantitative determination of binding constants.

Measurements were conducted in the following buffer: 10 mM HEPES, 150 mM NaCl, 1 mM MgCl_2_, 0.05–0.1% Tween-20, pH 7.4. The concentrations of all pure samples (Hb, aptamer, and control DNA) were fixed at 50 μM. For all mixed samples, the Hb concentration was fixed at 25 μM. The results are summarized in **Figure S3**.


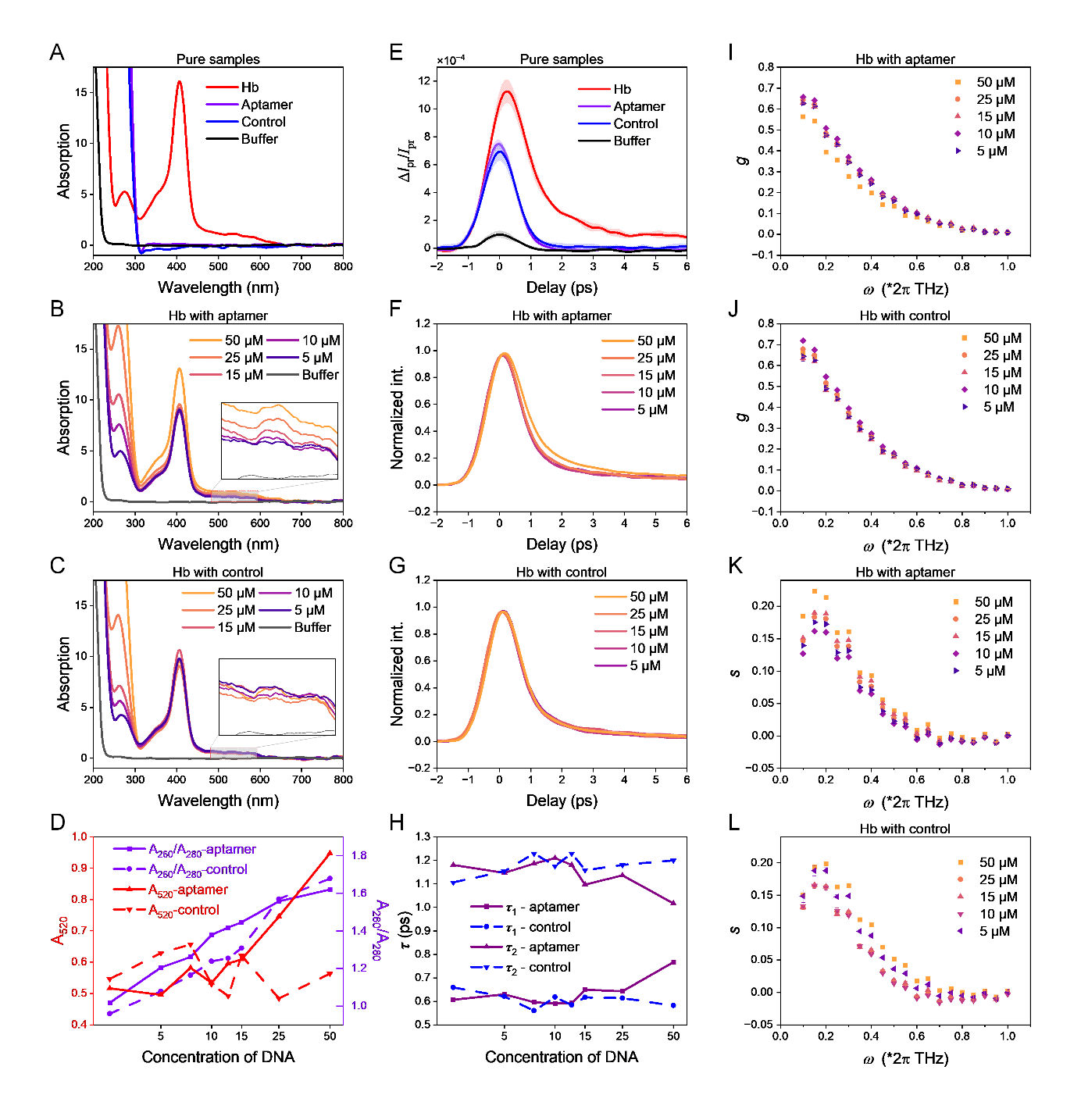


**Figure S3 | Interaction between hemoglobin and aptamer characterized by UV–Vis and transient absorption spectroscopy. (A)** UV–Vis absorption spectra of pure samples (Hb, aptamer, control DNA) and buffer. **(B–C)** UV-Vis absorption spectra of Hb mixed with increasing concentrations of aptamer (B) or control DNA (C). **(D)** Concentration dependence of A_520_ (red, left axis) and A_260_/A_280_ (purple, right axis) for aptamer (solid lines) and control (dashed lines).**(G)** Transient absorption kinetics of pure samples and buffer. **(E–F)** Normalized TA kinetics of Hb mixed with increasing concentrations of aptamer (E) or control DNA (F). **(H)** Concentration-dependent biexponential lifetimes (τ₁ and τ₂) extracted from TA kinetics for aptamer (solid symbols) and control (open symbols). **(I–L)** Phasor analysis of TA signals, where panels I and K correspond to aptamer, and J and L correspond to control DNA, respectively.

UV-Vis absorption spectra show that increasing aptamer concentration induces systematic changes in the Hb absorption band (e.g., A_520_​), whereas the control DNA produces minimal modulation (Figure S3B–D), suggesting that the observed spectral changes are not solely attributable to increased DNA content (tracked by A_260_​/A_280_).

In transient absorption measurements, the normalized kinetic traces reveal a concentration-dependent prolongation of the excited-state decay in the presence of aptamer, while control samples show much weaker or negligible lifetime changes (Figure S3F and S3G). Consistent with this trend, biexponential fitting indicates that the apparent decay components evolve in opposite directions with increasing aptamer concentration (τ_1_​ increases whereas τ_2_ decreases), accompanied by a reduced separation between τ_1_​ and τ_2_​ (Fig. S3H). This behavior is most reasonably interpreted as a redistribution between two relaxation channels or micro-environments rather than the emergence of a new decay pathway, suggesting that aptamer binding shifts the relative contributions of existing excited-state processes and renders the overall decay more single-exponential-like.

For phasor analysis, the presence of aptamer leads to a pronounced shift in the *g* component, whereas the control DNA produces negligible changes (Figure S3I and S3J). In contrast, the intrinsic symmetric Gaussian-like signal from DNA primarily contributes to a minor variation in the s component (Figure S3E), indicating that the aptamer-induced modulation is dominated by changes in the *g* component of the excited-state response rather than background absorption effects.

Notably, both the absorption amplitude and excited-state lifetime continue to evolve even when the aptamer concentration exceeds that of Hb, indicating that the interaction cannot be described by a simple 1:1 binding model. The non-monotonic dependence of lifetime parameters on DNA concentration further suggests that different binding configurations and/or binding-induced conformational modulation may exert distinct influences on the photophysical properties of Hb.

1. Lin, H.-I., Wu, C.-C., Yang, C.-H., Chang, K.-W., Lee, G.-B., and Shiesh, S.-C. (2015) Selection of aptamers specific for glycated hemoglobin and total hemoglobin using on-chip SELEX. Lab Chip, 15 (2), 486–494.
